# Diagnostic accuracy of transcranial sonography-magnetic resonance fusion imaging for Parkinson’s disease versus multiple system atrophy—Parkinsonian type

**DOI:** 10.3389/fneur.2026.1797261

**Published:** 2026-04-22

**Authors:** Chao Hou, Wei Zhang, Ji-zhu Xia, Hui Liu, Ming-xing Li, Wen He, Li-qing Peng

**Affiliations:** 1 Department of Ultrasound, the Affiliated Hospital, Southwest Medical University, Luzhou, Sichuan, China; 2 Department of Ultrasound, Beijing Tiantan Hospital, Capital Medical University, Beijing, China

**Keywords:** Parkinson’s disease, transcranial sonography, fusion imaging, substantia nigra, multiple system atrophy

## Abstract

**Introduction:**

Transcranial sonography-magnetic resonance (TCS-MR) fusion imaging shows promise in neurodegenerative diseases, yet current echogenicity assessment methods remain controversial, and its role in differentiating Parkinson’s disease (PD) from multiple system atrophy-parkinsonian type (MSA-P) remains unknown.

**Objectives:**

This study aims to evaluate the diagnostic and differential value of TCS-MR fusion imaging for PD versus MSA-P.

**Patients and methods:**

164 PD, 71 MSA-P, and 118 controls who underwent TCS-MR fusion imaging were prospectively enrolled. Substantia nigra hyperechogenicity (SNH) area was calculated. Three planes, designated as SN1, SN2 and SN3 from fusion images were analyzed using ImageJ for grayscale median and pixel count. ROC curves were employed to assess diagnostic and differential diagnostic power.

**Results:**

Statistically significant differences were observed among the three groups concerning SN grades, area of SNH, the ratio of the area of SNH to the midbrain area (S/M), echogenicity of the bilateral SN1, SN3, left SNH, and the maximum echogenicity of SN1, as well as the pixel count of bilateral SNH (*p <* 0.05). The maximum echogenicity of SN1 and left SN1 demonstrated the highest diagnostic and differential performance for PD, with AUC values of 0.86 and 0.82 (both *p <* 0.001). Fusion parameters outperformed the SNH area and S/M in both diagnosis and differential diagnosis of PD (Z = 3.84 and 3.71, *p <* 0.01).

**Conclusion:**

TCS-MR fusion imaging demonstrates superior diagnostic and differential performance for PD compared to traditional TCS measurements, suggesting its potential as a novel imaging technique in the diagnosis of PD.

## 1 Introduction

Parkinson’s disease (PD) is the second most prevalent neurodegenerative disorder threatening human health globally, with a high prevalence rates, significant disability, and substantial economic burden. It is projected that by 2050, there will be approximately 25.2 million PD patients worldwide (1). Consequently, accurate diagnosis of PD is crucial for developing effective treatment strategies. Currently, PD diagnosis relies on clinical manifestations, and the validation of modified clinical diagnostic criteria for PD, along with the testing of research criteria for prodromal PD, has enhanced diagnostic accuracy (2). Nevertheless, the clinical diagnosis of PD remains challenging due to the overlap of its features with other atypical Parkinsonism disorders, such as multiple system atrophy (MSA). MSA is pathologically characterized by glial cytoplasmic inclusions containing misfolded *α*-synuclein in oligodendrocytes. In the early stages of the disease, particularly the MSA-parkinsonian type (MSA-P), differentiation from PD is notably difficult, with clinical misdiagnosis rates reaching as high as 35% (3). Therefore, the development of novel methods to enhance PD diagnosis and its differential diagnosis from MSA-P has become a key focus in clinical practice.

Substantia nigra hyperechogenicity (SNH) is a distinctive feature of transcranial sonography (TCS) that cannot be identified using magnetic resonance imaging (MRI) or position emission tomography. Previous studies have demonstrated that SNH can effectively differentiate between PD and MSA (4, 5). However, TCS exhibits low spatial resolution, which hinders its ability to accurately delineate the structure and boundaries of the normal red nucleus and SN. The localization of SNH is somewhat reliant on the subjective assessment of the sonographer, resulting in poor result stability and diminished diagnostic efficacy. Fusion imaging achieves structural registration by leveraging the complementary strengths of various imaging techniques. Prior research has employed TCS-MR fusion imaging in the context of movement disorders (6, 7), addressing the challenges associated with TCS localization of deep brain nuclei. However, the researchers did not further investigate the efficacy of TCS-MR fusion imaging in the differential diagnosis of PD and MSA-P, and the proposed region of interest (ROI) segmentation method was subjectively dependent. Therefore, the present study focuses on a Chinese cohort to validate the feasibility of TCS-MR fusion imaging for the localization of midbrain nuclei, proposing a novel method for ROI segmentation and evaluating the diagnostic value of fusion imaging parameters for PD and their differential diagnosis with MSA-P.

## 2 Materials and methods

### 2.1 Study design and patient selection

Patients treated in our hospital’s Department of Dyskinesia and who underwent both brain MRI and TCS examinations were prospectively enrolled between November 2023 and October 2024. Eligible patients included those with PD or MSA-P aged 18 and older, provided they had clear brain imaging results. Two attending physicians, each with over 5 years of experience in movement disorders, making diagnoses for PD and MSA-P according to establishes standards (8, 9), or by a chief physician with more than 10 years of experience in cases of disagreement. Exclusion criteria: Parkinsonism resulting from other cerebral diseases, insufficient imaging results, poor temporal window, and any history of brain surgery. Additionally, age- and gender-matched volunteers without neurological diseases who visited the same medical center between March 2024 and November 2024 were recruited as control group. Demographic data were collected.

The study protocol was in accordance with the ethical standards of the 1974 Declaration of Helsinki and approved by our Hospital Regional Committee for Medical and Health Research Ethics (KY2022-015-04). Written informed consent was obtained from every patient before study inclusion.

### 2.2 Brain MRI examination

The protocol of brain MRI scanning and parameters of susceptibility-weighted imaging (SWI) were available in Supplementary information. Image analysis was conducted by two senior attending radiologists who were blinded to clinical data. The midbrain plane was identified, and the presence of bilateral “swallow-tail signs” on SWI sequence was examined in both the control and PD groups. Due to the asymmetric nature of PD onset, unilateral absence of the “swallow-tail sign” was deemed positive, irrespective of the presence of the sign on the contralateral side (10). The diagnostic results were compared between the two physicians, and cases with consistent findings were included in the diagnostic efficacy analysis.

### 2.3 TCS examination

All participants underwent TCS by using Aplio i900 (Canon, Japan) within 2 days following brain MRI. The TCS methodology adhered to the protocol described in our earlier study (11), details were available in Supplementary information. Two seasoned sonographers, each with over a decade of neuroimaging expertise and unaware of the clinical details, independently evaluated the SN grades. According to criteria defined by Bartova et al. (12), we defined SNH + as patchy hyperechogenicity appears in the SN with grading ≥ III (13). Calculated the area of SNH (aSNH) and midbrain, as well as ratio of aSNH to midbrain area (S/M). The calculation details followed guideline set by Bartova et al. (12), which were available in Supplementary information. Kappa consistency test and intraclass correlation coefficient (ICC) were used to access interobserver reliability for TCS measurement.

### 2.4 TCS-MR fusion imaging

After performing TCS scanning, the SWI data was uploaded to the ultrasound machine to execute fusion imaging. The scanning procedure adhered to the methodology outlined in our previous study (14), with further details available in the Supplementary information. MRI virtual navigation can assist in accurately locating the position and boundaries of midbrain nuclei in TCS. The images displayed during the examination included both TCS and MRI results, which could be viewed either side-by-side or in an overlapped format. Fusion imaging was conducted by a radiologist who was blinded to the clinical information and had undergone 1 month of training in fusion imaging training prior to the experiment.

### 2.5 ROI selection and grayscale median (GSM) analysis

**Figure 1:**
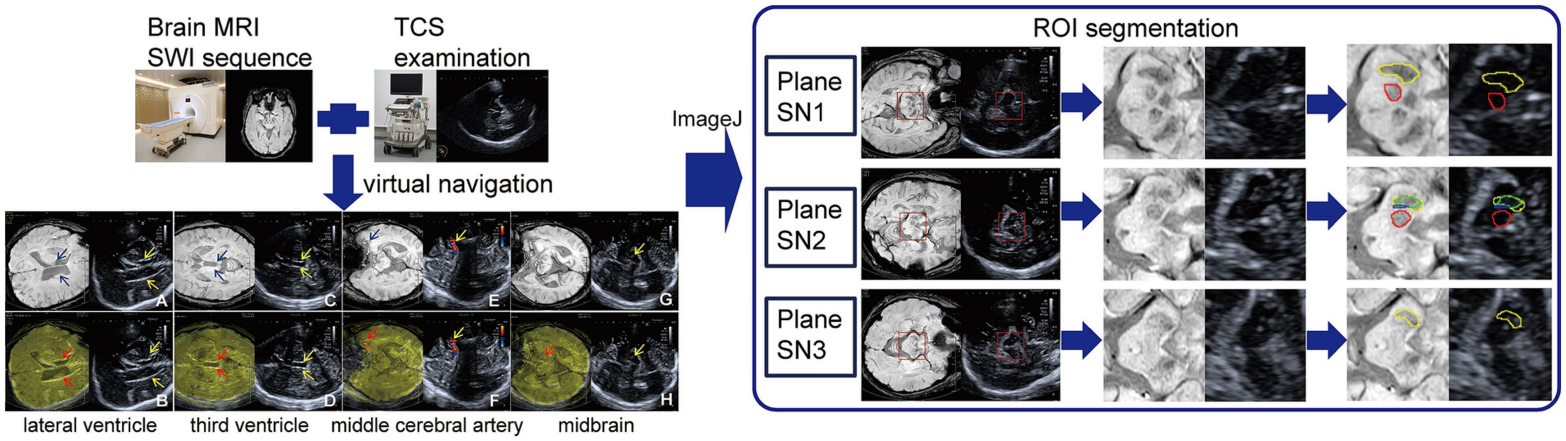
The workflow of TCS-MR fusion imaging, including representative planes of superimposition, and ROI segmentation. Left panel: the blue and yellow arrows indicated the lateral ventricle body (**A,B**), the third ventricle (**C,D**), and the left middle cerebral artery (**E,F**) on MRI and TCS, respectively, which were perfectly overlaid in fusion mode (red arrows). The yellow and red arrows in **G,H** indicated right substantia nigra hyperechogenicity (SNH) on TCS and fusion mode, respectively. Right panel: the yellow, red, green, and blue ROIs indicated boundaries of substantia nigra (SN), red nucleus, as well as SNH in the SN and dorsal bound of the SN, respectively. MRI, magnetic resonance imaging; SWI, susceptibility-weighted imaging; TCS, transcranial sonography; ROI, region of interest; plane SN1, the plane with the largest area of red nucleus on MRI; plane SN2, the plane with the largest area of substantia nigra hyperechogenicity; plane SN3, the plane where the red nucleus is barely identified or no longer visible.

A single researcher performed ROI segmentation and GSM analysis using ImageJ (National Institutes of Health, Bethesda, Maryland) with reference to previous literature (15). The side-by-side fusion images were split into a 640 × 960 matrix, after which the target ROIs on the MRI images were segmented and subsequently presented on TCS concurrently, using the relationships of coordinate positions. Three planes namely SN1, SN2, and SN3 were selected. The details and reason for choosing these three levels were available in Supplementary information. The ROIs of the red nucleus (RN) and SN in SN1, alongside SN, RN, and the hyperechogenic signal in SN2, and SN in SN3, were marked with different colors. During GSM analysis, the TCS image was converted to 8-bit format to standardize its’ background. Subsequently, the “intensity” option was selected to extract the mean pixel count and echogenicity values for each ROI. Figure 1 illustrates the entire process of fusion imaging, representative planes and ROI segmentation. The maximum echogenicity indices for SN1 were examined to ascertain which side displayed a higher echogenicity. After a two-week period, images from 20 participants were randomly chosen for repeat segmentation, ROIs for the SN and RN were accurately identified on the TCS image, yielding an ICC score of 0.84.

### 2.6 Statistical analysis

The determination of the sample size is available in Supplementary information. Measurement data was reported as mean ± standard deviation or median with interquartile range, while categories variables were indicated as percentages. In comparing group differences, unpaired independent *t*-test or one-way analysis of variance followed by Bonferroni’s corrected *post-hoc* comparisons were utilized for continuous variables with normal distributions. For continuous variables without normal distribution, Mann–Whitney U test or Kruskal–Wallis test was employed. The latter was followed by *post-hoc* comparisons using Dunn’s test with Bonferroni’s correction. And chi-square test was used for categorical variables. Receiver operating characteristic (ROC) curves were generated, and area under the curve (AUC), sensitivity, and specificity for diagnosing and differentiating PD were calculated. The Z test was used for comparing AUC differences. The Kappa consistency test and McNemar’s test were utilized for comparing the diagnostic efficacy of fusion imaging and MRI for PD. *p <* 0.05 indicated statistical significance. All statistical evaluations were carried out utilizing SPSS version 25.0 (IBM Corp., USA).

## 3 Results

### 3.1 Demographics characteristics

**Figure 2:**
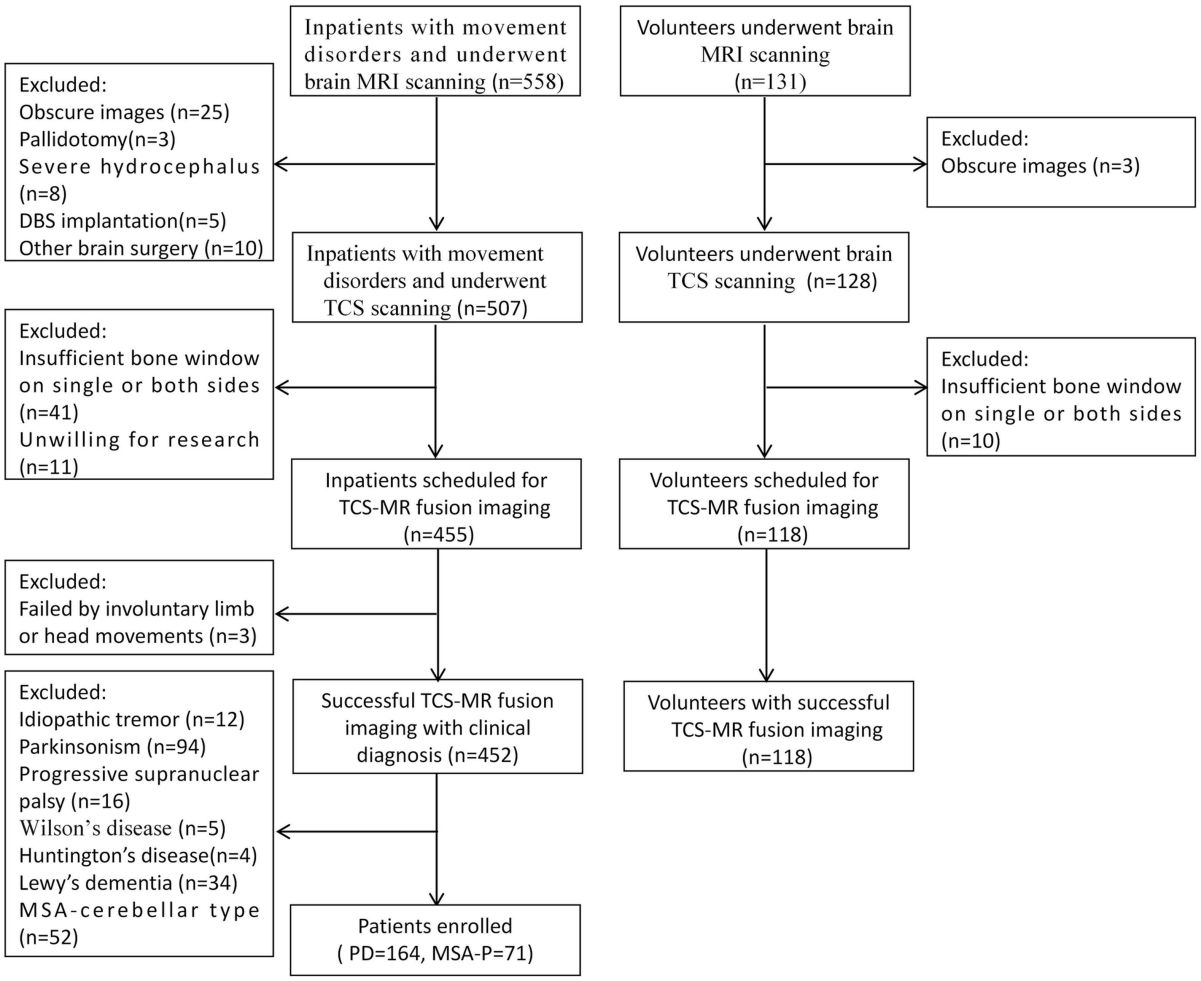
The flowchart diagram of patient selection.

Initially, 558 patients with movement disorders and 131 volunteers underwent brain MRI. According to the established inclusion and exclusion criteria, 573 participants underwent TCS-MR fusion imaging, of which 3 patients experienced failed image fusion due to involuntary limb or head movements. Therefore, the success rate of fusion imaging was 99.48%, 164 PD (63.70 ± 7.65 years, 65.24% male), 71 MSA-P (61.39 ± 7.65 years, 53.52% male), and 118 controls (61.69 ± 10.51 years, 70.34% male) were enrolled in this study. The average fusion time was 10.14 ± 0.30 min (Table 1). Figure 2 shows the patient selection flow diagram.

**Table 1:** Comparison of demographic and transcranial sonographic results of participants.

Parameters	HC (*n =* 118)	PD (*n =* 164)	MSA-P (*n =* 71)	*F/H/χ^2^*	*P*
Gender, male (*n*, %)	83 (70.34)	107 (65.24)	38 (53.52)	5.54	0.063^*^
Age (year)	61.69 ± 10.51	63.70 ± 9.69	61.39 ± 7.65	2.45	0.118^#^
BMI (Kg/m^2^)	25.51 ± 3.87	24.04 ± 3.12^a^	24.65 ± 3.04	646	0.002^#^
Area of midbrain (cm^2^)	5.33 ± 0.82	5.22 ± 0.73	4.99 ± 0.78^a^	4.36	0.013^#^
V3 width (mm)	4.84 ± 1.54	5.07 ± 1.66	5.38 ± 1.67	2.51	0.083^#^
SNH + (n, %)	50 (42.37)	119 (72.56)^aab^	37 (52.11)	27.16	<0.001^*^
left SNH + (*n*, %)	42 (35.59)	109 (64.02)^aabb^	29 (40.84)	29.83	<0.001^*^
right SNH + (*n*, %)	31 (26.27)	64 (46.34)^aabb^	20 (28.17)	14.35	0.001^*^
Left aSNH (cm^2^)	0 (00.18)	0.21 (00.30)^aabb^	0 (00.19)	33.95	<0.001^§^
Right aSNH (cm^2^)	0 (00.07)	0.05 (00.19)^ab^	0 (00.08)	14.22	0.001^§^
S/M (%)	1.67 (04.66)	5.69 (08.73)^aabb^	1.88 (05.14)	40.96	<0.001^§^
Maximum of aSNH (cm^2^)	0.07 (00.22)	0.24 (00.33)^aabb^	0.07 (00.21)	26.79	<0.001^§^
Total aSNH (cm^2^)	0.07 (00.28)	0.29 (00.48)^aabb^	0.09 (00.25)	38.70	<0.001^§^

F/H/*χ*^2^: F or H or *χ*^2^ statistic as appropriate to the test; *χ^2^ test; ^#^one-way ANOVA followed by Bonferroni’s *post-hoc* correction; ^§^Kruskal–Wallis test followed by *post-hoc* comparisons using Dunn’s test with Bonferroni’s correction; ^a^compared to HC, *p <* 0.05; ^aa^compared to HC, *p <* 0.001; ^b^compared to MSA-P, *p <* 0.05; ^bb^compared to MSA-P, *p <* 0.001; HC, healthy control; PD, Parkinson’s disease; MSA-P, multiple system atrophy-parkinsonian type; BMI, body mass index; V3, the third ventricle; SNH, substantia nigra hyperechogenicity; aSNH: area of substantia nigra hyperechogenicity; S/M, the ratio of the area of SNH to the midbrain area.

### 3.2 TCS findings

The Kappa and ICC values for TCS assessments were between 0.82 and 0.89. In the PD group, 119 cases (72.56%) exhibited SNH+, with 109 cases (64.02%) showing left SNH+, and 64 cases (46.34%) demonstrating right SNH+. The median (interquartile range) values for left aSNH, right aSNH, aSNHmax, total aSNH, and S/M were 0.21 (00.30) cm^2^, 0.05 (00.19) cm^2^, 0.24 (00.33) cm^2^, 0.29 (00.48) cm^2^, and 5.69 (08.73) %, respectively. Differences concerning SNH+, bilateral SNH + and aSNH, total aSNH, aSNHmax, and S/M ratio among the three groups were statistically significant (Table 1).

### 3.3 Fusion imaging results

In the left midbrain, the mean echogenicity indices of SN1, SN2, and SN3, as well as echogenicity and pixel count of SNH in PD group were significantly higher than those in controls and MSA-P group. In the right midbrain, the echogenicity values of SN1and SN3, and pixel count of SNH in the PD group were greater than those in controls group, while the pixel count of SN3 in both the PD and control groups exceeded those in the MSA-P group. Furthermore, the maximum echogenicity of SN1 in the PD group was significantly greater than that in the control group and the MSA-P group, and the maximum echogenicity of SN1 in the MSA-P in the MSA-P group was greater than that in the control group (Table 2).

**Table 2:** Comparison of transcranial sonography-magnetic resonance fusion imaging results of participants.

Parameters	HC (*n =* 118)	PD (*n =* 164)	MSA-P (*n =* 71)	*F/H/χ^2^*	*P*
Left side					
Pixel count of SN1	1191.21 ± 236.25	1196.79 ± 229.12	1138.80 ± 220.92	1.68	0.188^#^
GSM of SN1	18.31 ± 5.75	25.11 ± 6.87^aabb^	18.63 ± 5.75	49.52	<0.001^#^
Pixel count of SN2	1255.94 ± 257.23	1198.19 ± 248.37	1135.19 ± 245.89	2.25	0.109^#^
GSM of SN2	22.06 ± 6.98	28.45 ± 7.31^aabb^	22.40 ± 6.10	18.12	<0.001^#^
Pixel count of SN3	1067.87 ± 197.37	1076.73 ± 203.41	1028.31 ± 198.42	1.48	0.229^#^
GSM of SN3	20.16 ± 7.74	24.50 ± 11.32^aab^	20.53 ± 9.33	8.01	<0.001^#^
Pixel count of SNH	0 (0468.50)	421.00 (0702.00)^aabb^	0 (0.435.00)	26.37	<0.001^§^
GSM of SNH	0 (046.18)	39.47 (056.45)^ab^	0 (046.62)	17.08	<0.001^§^
Right side					
Pixel count of SN1	1185.46 ± 213.66	1179.55 ± 230.87	1120.46 ± 216.67	2.19	0.113^#^
GSM of SN1	16.77 ± 7.19	20.06 ± 9.12^aa^	19.23 ± 9.67	5.27	0.006^#^
Pixel count of SN2	1231.98 ± 231.16	1152.94 ± 245.17	1096.68 ± 261.54	2.62	0.077^#^
GSM of SN2	20.76 ± 7.99	23.07 ± 9.69	23.70 ± 9.73	1.085	0.341^#^
Pixel count of SN3	1059.96 ± 193.43	1056.02 ± 211.09^bb^	959.80 ± 204.24^aa^	6.53	0.002^#^
GSM of SN3	17.44 ± 6.66	21.09 ± 9.45^aa^	19.20 ± 9.68	6.13	0.002^#^
Pixel count of SNH	0 (0,134.50)	0 (0,388.25)^ab^	0 (0,166.00)	9.89	0.007^§^
GSM of SNH	0 (049.68)	0 (049.24)	0 (038.62)	3.54	0.170^§^
Maximum echogenicity of SN1	20.98 ± 6.28	27.16 ± 7.22^aabb^	22.72 ± 7.92	30.45	<0.001^#^

F/H/*χ*^2^: F or H or *χ*^2^ statistic as appropriate to the test; ^#^one-way ANOVA followed by Bonferroni’s *post-hoc* correction; ^§^Kruskal–Wallis test followed by *post-hoc* comparisons using Dunn’s test with Bonferroni’s correction; ^a^compared to HC, *p <* 0.05; ^aa^compared to HC, *p <* 0.001; ^b^compared to MSA-P, *p <* 0.05; ^bb^compared to MSA-P, *p <* 0.001; HC, healthy control; PD, Parkinson’s disease; MSA-P, multiple system atrophy-parkinsonian type; SN, substantia nigra; GSM, grayscale median; SNH: substantia nigra hyperechogenicity.

A comparison of the SN parameters on the left and right sides revealed that, in the PD group, both the aSNH and the echogenicity of SN1, SN2, and SN3 on the right side were lower than those on the left side. However, these statistical differences were only observed in the SN3 pixel count within the MSA-P group and in the SN3 echogenicity in the control group (Supplementary Table 1).

### 3.4 Diagnostic and discriminable performance of TCS and fusion imaging

ROC curves showed that among the traditional measurement indicators of TCS, the S/M ratio and total aSNH exhibited the greatest diagnostic and differential diagnostic performance for PD, with respective AUC values of 0.70 (*p <* 0.001, 95% [confidence interval, *CI*]: 0.64–0.76) and 0.69 (*p <* 0.001, 95% *CI*: 0.62–0.76).

**Figure 3:**
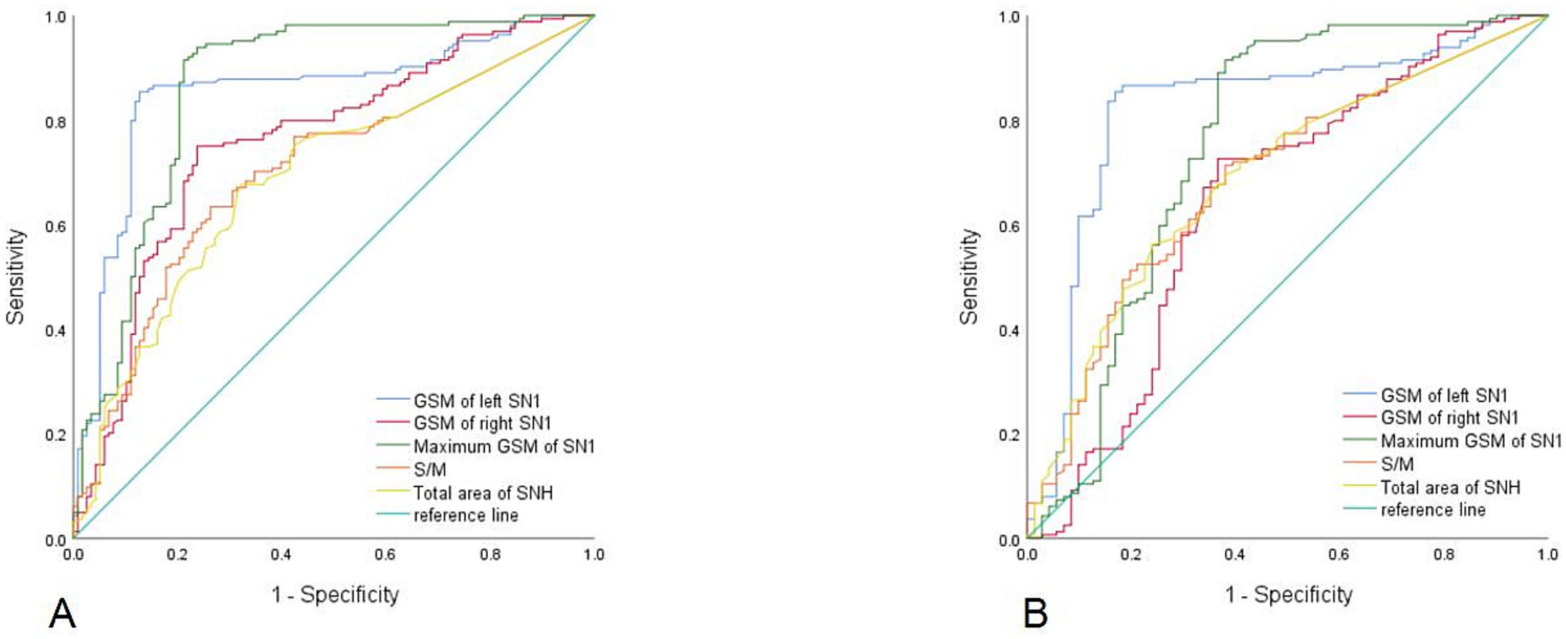
The receiver operating characteristic curves to identifying Parkinson’s disease **(A)** and differentiating Parkinson’s disease from multiple system atrophy-parkinsonian type **(B)**. GSM, grayscale median; SN1, substantia nigra in the plane exhibiting the largest red nucleus area on MRI; S/M, area of SNH/area of midbrain; SNH, substantia nigra hyperechogenicity.

Among the fusion parameters, the maximum echogenicity of SN1 had the highest diagnostic power for PD (AUC = 0.86, *p <* 0.001, 95% *CI*: 0.0.82–0.91), with a cut-off value of 21.70 resulting in high sensitivity (97.5%) and moderate specificity (78.8%) (Figure 3A). Furthermore, echogenicity of left SN1 performed well in distinguishing PD from MSA-P, yielding an AUC of 0.82 (*p <* 0.001, 95% *CI*: 0.76–0.89), with a sensitivity of 85.4% and specificity of 83.1% (Figure 3B). Table 3 summarizes the AUC, cut-off value, sensitivity, and specificity of each parameter for PD diagnosis and differential diagnosis.

**Table 3:** Diagnostic and differential diagnostic performance of parameters for Parkinson’s disease.

PD diagnosis	AUC (*P*)	95%*CI*	Cut-off value	Sensitivity (%)	Specificity (%)
S/M	0.70 (<0.001)	0.64,0.76	4.3%	63.4	73.5
GSM of left SN1	0.79 (<0.001)	0.74,0.85	21.73	78.0	83.8

Differentiating PD from MSA	AUC (*P*)	95% *CI*	Cut-off value	Sensitivity (%)	Specificity (%)
Total aSNH	0.69 (<0.001)	0.62, 0.76	0.26	56.1	76.1
GSM of left SN1	0.79 (<0.001)	0.72, 0.85	21.64	78.0	84.5

PD, Parkinson’s disease; AUC, area under the curve; CI, confidence interval; S/M, the ratio of the area of SNH to the midbrain area; GSM, grayscale median; SN, substantia nigra; MSA-P, multiple system atrophy-parkinsonian type; aSNH: area of substantia nigra hyperechogenicity.

The Z test results revealed that the AUC differences between maximum echogenicity of SN1 and S/M (Z = 3.84, *p <* 0.01) in diagnosing PD, as well as between left SN1 echogenicity and total aSNH (Z = 3.71, *p <* 0.01) in differential diagnosing PD were statistically significant.

### 3.5 Comparison of diagnostic power of “swallow-tail sign” and fusion imaging for PD

**Figure 4:**
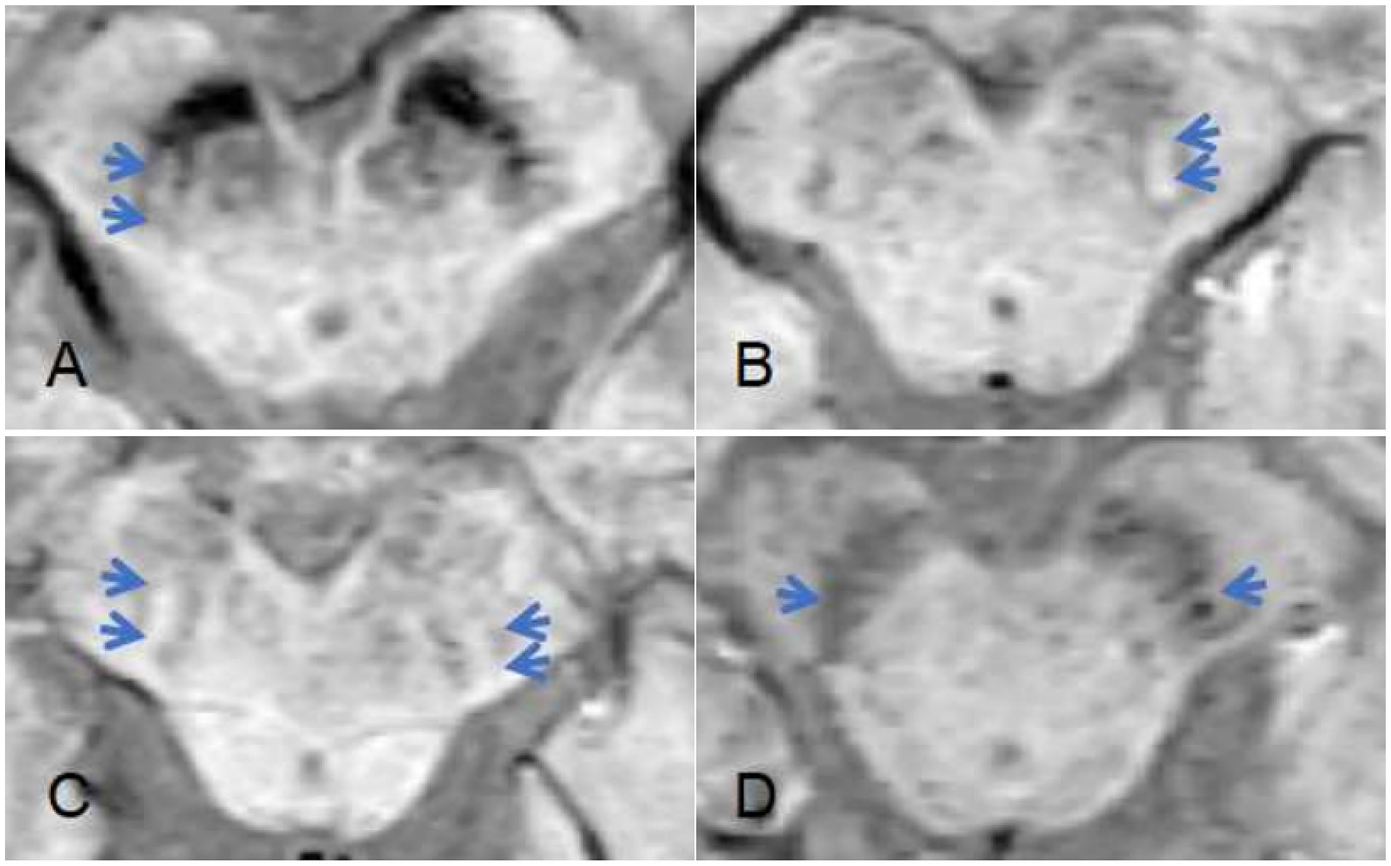
Representative cases of bilateral swallow-tail sign on susceptibility-weighted imaging sequence. **(A)** The presence of the swallow-tail sign on the right side; **(B)** the presence of the swallow-tail sign on the left side; **(C)** the presence of the swallow-tail sign on the bilateral sides; **(D)** absence of swallow-tail sign on both sides.

Two radiologists reached an agreement on the MRI diagnostic results in 141 PD cases and 108 control cases. Therefore, these 141 PD cases and 108 controls were used for comparing the diagnostic ability of “swallow-tail sign” and fusion imaging (Figure 4). The sensitivity and specificity for diagnosing PD using “swallow-tail sign” were found to be 81.56 and 59.26%, respectively. When applying a cut-off value of 21.70 for the maximum echogenicity of SN1, generating a sensitivity of 90.2% and a specificity of 77.8% (Table 4). The Kappa value was 0.143, indicating a poor consistency in the diagnostic results between the two methods. McNemar’s test results showed that the detection rate of PD via the maximum echogenicity of SN1 was significantly higher than that of the “swallow-tail sign” (detection rates of 90.8% versus 80.9%, *p* = 0.016).

**Table 4:** Comparison of diagnostic power of swallow-tail sign and GSM of left SN1 for Parkinson’s disease.

Parameters	Parkinson’s disease	Healthy control	*χ^2^*	*P*
Swallow-tail sign positive	115	44	44.15	<0.001
	26	64		
GSM of left SN1 below cut-off value	109	17	92.73	<0.001
	32	91		

*χ*^2^: *χ*^2^ statistic as appropriate to chi-square test; GSM, grayscale median; SN, substantia nigra.

## 4 Discussion

Due to the partial overlapping clinical symptoms between MSA-P and PD, the differential diagnosis of these two conditions poses significant challenges. Building upon our previous research (14), this study successfully achieved segmentation of the ROI on TCS-MR fusion images by utilizing positional coordinate relationships, and compared the efficacy of fusion imaging parameters against conventional TCS for the diagnosis of PD and its differentiation from MSA-P.

In this study, the prevalence of SNH + in the control cohort was found to be 42.37%, which is higher than the findings reported by Behnke et al. (16) but lower than those of Hagenah et al. (17). The difference in the proportion of SNH + between the control and MSA-P groups was not significant. This discrepancy may be attributed to variations in participants age, as the mean age of volunteers in this study was 61.69 ± 10.51 years, and SNH tends to increase with age (17). Additionally, the differing definitions of SNH + contribute to the observed high proportion in our control group. In this study, a visual assessment method was employed to grade SN, whereas other studies defined SNH + as a unilateral area of SNH measuring ≥ 0.20cm^2^ (18, 19). Furthermore, slight difference in anatomical structures still exist among different ethnicities.

A meta-analysis indicates that the sensitivity (ranging from 48.7 to 93.4%) and specificity (ranging from 55.2 to 92.0%) of TCS-identified SNH in differentiating PD from essential tremor exhibits significant variability across different cutoff values (0.14–0.25 cm^2^) (20), indirectly indicates that the diagnostic results of TCS exhibit significant heterogeneity. The imaging quality of TCS is affected by both acoustic and electronic noise, coupled with the use of low-frequency probes, resulting in compromise between depth of penetration and spatial resolution, leading to a limited resolution of TCS for specific structures. Consequently, the anatomical localization of the midbrain nucleus relies heavily on the subjective judgement of the sonographer, which diminishes the diagnostic performance for PD. Ultrasound-MR fusion imaging leverages the high spatial resolution of MRI alongside the dynamic real-time characteristics of ultrasound and has been successfully applied in the diagnosis and treatment of various diseases within the urinary, digestive, and reproductive system (21). In our prior study, TCS-MR fusion imaging was innovatively utilized in neurosurgical tumors, and in conjunction with contrast-enhanced ultrasound, it more accurately delineated the boundaries of gliomas and differentiated the edema zone (22). However, the potential application of TCS-MR fusion imaging in movement disorders remains largely unexplored.

Research teams lead by Skoloudik D and Walter U have conducted a series of studies applying TCS-MR fusion imaging in conjunction with digitized analysis for movement disorders (7, 23–25). The SN echogenicity of early-onset PD was significantly higher than that in control subjects (24), which aligns with the findings of this study. However, the registration of TCS with MRI structures and the selection of ROI are critical for subsequent digitized analysis. In the study conducted by Skoloudik D et al. (24), TCS and MRI images were automatically registered using a virtual navigation system. An elliptical ROI with an area of 50 mm^2^ was manually placed on the fused images based on the empirical knowledge regarding the anatomical location of the SN. Nevertheless, this fixed-size elliptical ROI does not precisely represent the actual condition of the nucleus, as both the signal and volume of the nucleus may vary with disease progression and drug treatment (26). Additionally, the manual placement of the ROI is heavily dependent on assessment of sonographer.

This study stands out in the following merits compared to previous studies applying TCS-MR fusion imaging in PD (23–25). Firstly, we have a significant advantage concerning population size, as our study included 164 patients with PD, 71 MSA-P, and 118 controls, whereas prior study comprised only 16 to 24 patients in each group. Secondly, the SWI sequence was selected for image fusion due to its ability to clearly display the shape and boundaries of the SN (in most cases) and RN. At the same time, the swallow-tail sign observed on SWI is regarded as a sensitive imaging marker for PD (26), allowing for future exploration of the association between SNH and nigrosome-1; specifically, the absence of the swallow-tail sign indicates the loss of the hyperintense signal associated with nigrosome-1 on SWI (27), which is a manifestation of iron deposition and thought to be related to the echogenicity changes of SN in PD patients (28). Thirdly, during the image analysis, we converted the side-by-side image into 640 × 960 matrix, which effectively splits the image into two halves. We then used ImageJ to select the ROIs on the MRI images, enabling the creation of precise copies on the TCS images based on the coordinate relationship to the ROIs on the MRI images. The calculations of pixel count and GSM can effectively represent the size and echo value of each ROI.

The findings indicated that the maximum echogenicity of SN1 and echogenicity of left SN1 exhibited greater diagnostic and differential diagnostic power in PD than traditional TCS parameters like SNH area and S/M ratio. The calculation of the SN echogenicity is performed by outlining the contour of the SN on the same plane of the SWI sequence, with quantified changes in its gray value reflecting alterations in the echo within the SN. On the contrary, the delineation of the SNH area, even by experienced ultrasound physicians, relies on subjective judgment in making the anatomical location of the SN and may not truly display the boundaries between the SN and adjacent nuclei. Furthermore, whether the outlined “SNH” is exclusively located within the SN remains a contentious issue. Therefore, the digitized analysis of the SN utilizing TCS-MR fusion imaging may be more effective than manual identification of the SNH, potentially serving as an alternative method to assist in the diagnosis and differentiation diagnosis of PD. This aligns with previous findings (25). In addition, a recent study employed shear wave elastography to assess the elasticity of SN, indicated SN elasticity values are a promising classification biomarker for PD (29). In the future, the integration of MR elastography and shear wave elastography within the framework of fusion imaging may enhance the diagnostic specificity for PD.

Furthermore, the changes in echogenicity were more pronounced in the SN1 than in the SN3 on both sides, with the selected SN1 and SN3 planes matching to the rostral and caudal levels, respectively, as previously reported (30). These results demonstrate that echogenic changes in the SN of PD patients occur more prominently in the rostral region than in the caudal regions, which sheds more light on the lack of correlation between quantitative susceptibility mapping results and TCS findings in PD patients (31), as the former shows more significant differences at the caudal level (30), while the latter is more obvious at the rostral level, implying that quantitative susceptibility mapping and SNH may reflect different pathological states of iron deposition in PD.

To be noted, the difference in SN parameters between the left and right sides in PD group were more obvious than those in the control and MSA-P groups. Similar findings were reported in the study by Iranzo et al. (32). This phenomenon may be attributed to the asymmetric loss of dopaminergic neurons in the cerebral hemispheres of patients with PD, where neurodegeneration typically begins in the dominant hemisphere. All participants in this study were right-handed, which is associated with left-hemispheric dominance in nigrostriatal dysfunction. This asymmetrical neurodegeneration may initially weaken the nigrostriatal system of the dominant hemisphere (32), indicating that right-handedness could enhance susceptibility to PD pathology through variations in the activation levels of basal ganglia motor circuits, with the left nigrostriatal system displaying higher baseline activity (33).

The disappearance of swallow-tail sign on SWI sequence serves as a significant imaging marker for PD. In order to compare the diagnostic power of fusion imaging and SWI for PD, 141 PD cases and 108 controls with both imaging evaluation were analyzed. Results revealed that the maximum grayscale value of SN1 has a higher detection rate than the swallow-tail sign. Nigrosome-1 is the first structure to be affected during the onset of PD, and the swallow-tail sign indicates the high signal of nigrosome-1 located at the caudal level of the SN on the SWI sequence, the disappearance of swallow-tail sign represents local iron deposition (34). However, as previously mentioned, iron deposition is only one potential mechanism contributing to elevated SN echogenicity. Given the complexity and heterogeneity of PD pathogenesis, imaging features indicative of iron deposition alone may not adequately reflect all pathological processes associated with PD. Therefore, we propose that changes in SN GSM may better represent the disease status of PD than the swallow-tail sign.

Limitations of this study need to be mentioned. The primary issue is the absence of neuropathological confirmation of the diagnosis in all patients, which is the current challenge in the management of PD. Nevertheless, our medical center, as a National Neurology Centre in China, provides a certain degree of reliability regarding clinical diagnoses. Secondly, in the image registration process, both automatic and manual fine-tuning were utilized; however, this approach may still lead to a positional deviation of 1–2 mm. Nevertheless, a positional difference of < mm during image fusion process is regarded as the optimal registration outcome (7). However, potential image distortion issues during registration process must still be taken into account, as they may introduce bias in subsequent measurement. Thirdly, the main objective of this study was to compare the efficacy of fusion imaging for diagnosing and differentiating PD. Consequently, we did not analyze the spatial location of SNH and the correlation between fusion imaging features and clinical characteristics. However, we intend to present this aspect of the results in another manuscript (14). Furthermore, approximately 10% of subjects exhibit insufficient bilateral temporal window, which restricts the applicability of TCS/MR fusion imaging within this cohort.

## 5 Conclusion

The maximum echogenicity of SN1 and echogenicity of left SN1 based on TCS-MR fusion imaging demonstrates higher diagnostic and differential power than traditional metrics such as SNH area and S/M. The proposed ROI segmentation method accurately and reliably captures differences in the SN, thereby enabling further analysis of the spatial changes in SN echogenicity with the progression of PD.

## Data Availability

The raw data supporting the conclusions of this article will be made available by the authors, without undue reservation.

## References

[1] Su D Cui Y He C Yin P Bai R Zhu J . Projections for prevalence of Parkinson's disease and its driving factors in 195 countries and territories to 2050: modelling study of global burden of disease study 2021. BMJ. (2025) 388:e080952. doi: 10.1136/bmj-2024-080952, 40044233 PMC11881235

[2] Heinzel S Berg D Gasser T Chen H Yao C Postuma RB . Update of the MDS research criteria for prodromal Parkinson's disease. Mov Disord. (2019) 34:1464–70. doi: 10.1002/mds.27802, 31412427

[3] Adler CH Beach TG Hentz JG Shill HA Caviness JN Driver-Dunckley E . Low clinical diagnostic accuracy of early vs advanced Parkinson disease: clinicopathologic study. Neurology. (2014) 83:406–12. doi: 10.1212/WNL.0000000000000641, 24975862 PMC4132570

[4] Wang LS Yu TF Chai B He W. Transcranial sonography in differential diagnosis of Parkinson disease and other movement disorders. Chin Med J. (2021) 134:1726–31. doi: 10.1097/CM9.0000000000001503, 34238849 PMC8318650

[5] Walter U Niehaus L Probst T Benecke R Meyer BU Dressler D. Brain parenchyma sonography discriminates Parkinson's disease and atypical parkinsonian syndromes. Neurology. (2003) 60:74–7. doi: 10.1212/WNL.60.1.74, 12525721

[6] Maskova J Skoloudik D Burgetova A Fiala O Brůha R Záhoráková D . Comparison of transcranial sonography-magnetic resonance fusion imaging in Wilson's and early-onset Parkinson's diseases. Parkinsonism Relat Disord. (2016) 28:87–93. doi: 10.1016/j.parkreldis.2016.04.031, 27147115

[7] Walter U Müller J-U Rösche J Kirsch M Grossmann A Benecke R . Magnetic resonance-transcranial ultrasound fusion imaging: a novel tool for brain electrode location. Mov Disord. (2016) 31:302–9. doi: 10.1002/mds.26425 26362398

[8] Gilman S Wenning GK Low PA Brooks DJ Mathias CJ Trojanowski JQ . Second consensus statement on the diagnosis of multiple system atrophy. Neurology. (2008) 71:670–6. doi: 10.1212/01.wnl.0000324625.00404.15, 18725592 PMC2676993

[9] Postuma RB Berg D Stern M Poewe W Olanow CW Oertel W . MDS clinical diagnostic criteria for Parkinson's disease. Mov Disord. (2015) 30:1591–601. doi: 10.1002/mds.26424, 26474316

[10] Wang N Liu XL Li L Zuo CT Wang J Wu PY . Screening for early-stage Parkinson's disease: swallow tail sign on MRI susceptibility map-weighted images compared with PET. J Magn Reson Imaging. (2021) 53:722–30. doi: 10.1002/jmri.27386, 33096586

[11] Hou C Yang F Li S Ma HY Li FX Zhang W . A nomogram based on neuron-specific enolase and substantia nigra hyperechogenicity for identifying cognitive impairment in Parkinson's disease. Quant Imaging Med Surg. (2024) 14:3581–92. doi: 10.21037/qims-23-1778, 38720848 PMC11074765

[12] Bartova P Skoloudik D Bar M Ressner P Hlustik P Herzig R . Transcranial sonography in movement disorders. Biomed Pap Med Fac Univ Palacky Olomouc Czech Repub. (2008) 152:251–8. doi: 10.5507/bp.2008.039, 19219216

[13] Fan Y Ma J Yang D Li X Liang K She Z . Clinical findings of hyperechoic substantia nigra in patients with Parkinson's disease. Eur J Neurosci. (2024) 59:2702–14. doi: 10.1111/ejn.16308, 38469656

[14] Hou C Zhang W Li HB Li S Nie F Wang XM . Spatial variations and precise location of substantia nigra hyperechogenicity in Parkinson's disease using TCS-MR fusion imaging. NPJ Parkinsons Dis. (2025) 11:78. doi: 10.1038/s41531-025-00910-7, 40240329 PMC12003750

[15] Yu JL Wiemken A Schultz SM Keenan BT Sehgal CM Schwab RJ. A comparison of ultrasound echo intensity to magnetic resonance imaging as a metric for tongue fat evaluation. Sleep. (2022) 45:zsab295. doi: 10.1093/sleep/zsab295, 34963001 PMC8842321

[16] Behnke S Double KL Duma S Broe GA Guenther V Becker G . Substantia nigra echomorphology in the healthy very old: correlation with motor slowing. NeuroImage. (2007) 34:1054–9. doi: 10.1016/j.neuroimage.2006.10.010, 17141529

[17] Hagenah J Konig IR Sperner J Wessel L Seidel G Condefer K . Life-long increase of substantia nigra hyperechogenicity in transcranial sonography. NeuroImage. (2010) 51:28–32. doi: 10.1016/j.neuroimage.2010.01.112, 20152909

[18] Planas-Ballve A Rios J Gea M Rabaneda-Lombarte N Ispierto L Grau L . Substantia nigra hyperechogenicity and brain ventricular size as biomarkers of early dementia with Lewy bodies. Alzheimer's Res Ther. (2024) 16:227. doi: 10.1186/s13195-024-01590-w, 39407323 PMC11475835

[19] Zhu S Shi Y Chen Z Long Z Wan L Chen D . The characteristic and biomarker value of transcranial sonography in cerebellar ataxia. Ann Clin Transl Neurol. (2024) 11:2100–11. doi: 10.1002/acn3.52131, 38924300 PMC11330234

[20] Heim B Peball M Hammermeister J Djamshidian A Krismer F Seppi K. Differentiating Parkinson's disease from essential tremor using transcranial sonography: a systematic review and Meta-analysis. J Parkinsons Dis. (2022) 12:1115–23. doi: 10.3233/JPD-213012, 35180133 PMC9198761

[21] Lin Y Yilmaz EC Belue MJ Harmon SA Tetreault J Phelps TE . Evaluation of a cascaded deep learning-based algorithm for prostate lesion detection at Biparametric MRI. Radiology. (2024) 311:e230750. doi: 10.1148/radiol.230750, 38713024 PMC11140533

[22] Wu DF He W Lin S Han B Zee CS. Using real-time fusion imaging constructed from contrast-enhanced ultrasonography and magnetic resonance imaging for high-grade glioma in neurosurgery. World Neurosurg. (2019) 125:e98–e109. doi: 10.1016/j.wneu.2018.12.215, 30677585

[23] Skoloudik D Bartova P Maskova J Dušek P Blahuta J Langová K . Transcranial sonography of the insula: digitized image analysis of fusion images with magnetic resonance. Ultraschall Med. (2016) 37:604–8. doi: 10.1055/s-0042-111822 27486795

[24] Skoloudik D Maskova J Dusek P Blahuta J Soukup T Burgetová A . Digitized image analysis of insula echogenicity detected by TCS-MR fusion imaging in Wilson's and early-onset Parkinson's diseases. Ultrasound Med Biol. (2020) 46:842–8. doi: 10.1016/j.ultrasmedbio.2019.12.013, 31924422

[25] Kozel J Skoloudik D Ressner P Michalčová P Dušek P Hanzlíková P . Echogenicity of brain structures in Huntington's disease patients evaluated by transcranial sonography - magnetic resonance fusion imaging using virtual navigator and digital image analysis. Ultraschall Med. (2023) 44:495–502. doi: 10.1055/a-2081-1635 37224875 PMC11928295

[26] Jokar M Jin Z Huang P Wang Y Zhang Y Li Y . Diagnosing Parkinson's disease by combining neuromelanin and iron imaging features using an automated midbrain template approach. NeuroImage. (2023) 266:119814. doi: 10.1016/j.neuroimage.2022.119814, 36528314

[27] Bae YJ Kim JM Sohn CH Choi JH Choi BS Song YS . Imaging the substantia nigra in Parkinson disease and other parkinsonian syndromes. Radiology. (2021) 300:260–78. doi: 10.1148/radiol.2021203341, 34100679

[28] Luyken AK Lappe C Viard R Löhle M Kleinlein HR Kuchcinski G . High correlation of quantitative susceptibility mapping and echo intensity measurements of nigral iron overload in Parkinson's disease. J Neural Transm (Vienna). (2025) 132:407–17. doi: 10.1007/s00702-024-02856-1, 39485510 PMC11870917

[29] Xu X Guo Y Xiong Y Min Z Xue Z Deng Y . Substantia Nigra elasticity measurement using transcranial shear wave Elastography: a potential biomarker for Parkinson's disease. Mov Disord. (2025) 40:2169–76. doi: 10.1002/mds.30289, 40616442

[30] Lee H Cho H Lee MJ Kim TH Roh J Lee JH. Differential effect of Iron and myelin on susceptibility MRI in the substantia Nigra. Radiology. (2021) 301:682–91. doi: 10.1148/radiol.2021210116, 34609198

[31] Ahmadi SA Bötzel K Levin J Maiostre J Klein T Wein W . Analyzing the co-localization of substantia nigra hyper-echogenicities and iron accumulation in Parkinson's disease: a multi-modal atlas study with transcranial ultrasound and MRI. NeuroImage: Clinical. (2020) 26:102185. doi: 10.1016/j.nicl.2020.102185, 32050136 PMC7013333

[32] Iranzo A Stefani A Ninerola-Baizan A Stokner H Serradell M Vilas D . Left-hemispheric predominance of nigrostriatal deficit in isolated REM sleep behavior disorder. Neurology. (2020) 94:e1605–13. doi: 10.1212/WNL.0000000000009246, 32161031

[33] Garrido A Iranzo A Stefani A Serradell M Muñoz-Lopetegi A Marrero P . Lack of asymmetry of nigrostriatal dopaminergic function in healthy subjects. Mov Disord. (2020) 35:1072–6. doi: 10.1002/mds.28019, 32141653

[34] Lim SJ Suh CH Shim WH Kim SJ. Diagnostic performance of T2* gradient echo, susceptibility-weighted imaging, and quantitative susceptibility mapping for patients with multiple system atrophy-parkinsonian type: a systematic review and meta-analysis. Eur Radiol. (2022) 32:308–18. doi: 10.1007/s00330-021-08174-4, 34272590

