# Supplementary material for: Diagnostic accuracy of transcranial sonography-magnetic resonance fusion imaging for Parkinson’s disease versus multiple system atrophy—Parkinsonian type
Source: Front Neurol. 2026 Apr 22;17:1797261. doi: 10.3389/fneur.2026.1797261 (PMC13143536; doi:10.3389/fneur.2026.1797261)
Supplement: Supplementary file 1 [file Supplementary_file_1.docx]

**Supplementary Information**

1. **Details of brain MRI test and scanning parameters of SWI sequence**

Brain magnetic resonance (MR) images were obtained using a 3T MRI scanner (Ingenia CX, Philips, Best, The Netherlands). Scanning sequences included three-dimensional T1-weighted imaging, axial T2-weighted imaging, axial fluid-attenuated inversion recovery, and axial susceptibility-weighted imaging (SWI). Axial sections were taken between the anterior and posterior commissures, aligned parallel to the standard reference plane, covering the cranial vault to the foramen magnum occipitalis. Scanning parameters of SWI sequence: repetition time: 29.98 ms, echo time: 20 ms, field of view: 220x180 mm, matrix: 384x256, slice thickness: 2.5 mm, spacing between slices 1.25mm, flip angle: 15°. Prior to scanning, an automatic tracking positioner was affixed to the forehead with its position on the head manually marked to facilitate subsequent fusion imaging. After scanning, the SWI sequence was selected and exported in DICOM format.

1. **Details of transcranial sonography (TCS) examination and image analysis**

TCS test was utilized by an Aplio i900 ultrasound machine (Canon, Japan) equipped with an I6SX1 probe and conducted by a skilled sonographer. The center frequency was 2.6MHz, scanning depth was 14-16cm, dynamic range was 45-55 dB, mechanical index was 1.1, frame rate was 45, wide view (off). Image brightness and gain compensation were adjusted according to the patient’s condition.

The grades of substantia nigra were assessed according to the guidelines set by Bartova P^[1]^, with a grading of ≥III signifying substantia nigra hyperechogenicity+ (SNH+). The area of SNH (aSNH) was quantified by outlining and measuring hyperechoic signals in the ipsilateral temporal window. In cases where bilateral hyperechoic signals were present, they were noted individually; aSNHmax corresponded to the larger aSNH of the two sides or was considered when one side had inadequate bone window. The ratio of aSNH to midbrain area (S/M) was determined using the equation: = [left aSNH + right aSNH) / area of midbrain] * 100%.

1. **Protocol of TCS-MR fusion imaging**

The ultrasound machine (Aplio i900, Canon, Japan) integrated the Virtual Navigator procedures, which included a position sensing unit affixed to the ultrasound device, a magnetic field transmitter, and a sensor connected to the probe via a designated holder. Each patient was registered using his hospitalization number to retrieve the corresponding information linked to the MRI data. Virtual navigation mode was then activated, allowing both the MRI and TCS images to be displayed simultaneously. Initially, the patient’s head position was recorded by identifying five common reference points using the tracking system, typically resulting in a nearly identical match between the patient’s head position (aligned with the real-time TCS image coordinates) and the MRI dataset. Subsequently, the MRI and TCS images merged accurately through a fine-tuning process. This involved manually dragging the frozen MRI and superimposed TCS images on the monitor towards each other. Careful focus was given to the precise alignment of the structures clearly discernible in both MRI and TCS, including the anterior horn of the lateral ventricles, the third ventricle, thalamus, pineal gland, midbrain, Willis’s circle, and aqueduct. This process was repeated until optimal superimposition was achieved, maintained by continuous position tracking using the two sensors placed on the patient’s head and the ultrasound transducer, ensuring a signal quality of 9-10 points. During the real-time TCS examination, the MRI volume automatically scrolled to exhibit matching planes with the Virtual Navigator, while the patient’s head remained still throughout the procedure. The images displayed during the examination consisted of both TCS and MRI results, which could be viewed either side-by-side or overlapped. Dynamic video capture was performed from the basal ganglia level to the pontine level for subsequent analysis.

1. **Details and reason for choosing SN1, SN2, and SN3 for ROI segmentation and grayscale analysis**

**Details:** three planes on TCS corresponding to MRI were chosen based on anatomical landmarks in fusion images: the plane exhibiting the largest red nucleus (RN) area on MRI, the plane showcasing the most substantial aSNH, and the plane where the RN was minimally visible, designated as plane SN1, SN2, and SN3, respectively.

**Reason**: first, the planes SN1 and SN3 on MRI correspond to the rostral and caudal levels of substantia nigra^[2]^, respectively. This correspondence facilitates comparative analysis and discussion regarding the swallow-tail sign observed in the SWI sequence. Second, this selection lays on the groundwork for the group’s forthcoming exploration on the spatiotemporal variation of substantia nigra hyperechogenicity.

**5. Calculation of sample size:**

The determination of the sample size was based on the primary aim of assessing the procedure’s feasibility, drawing references from a prior study^[3]^. A total of 45 participants was established with the assumption of a 95% success rate, a confidence level of 95%, and a 5% margin of error. Including 164 participants in the Parkinson’s disease group and 71 in the multiple system atrophy-parkinsonian type group ensured adequate power to handle potential missing data arising from limited bone windows.

**Reference**

[1] Bartova P., Skoloudik D., Bar M. et al (2008) Transcranial sonography in movement disorders. Biomed Pap Med Fac Univ Palacky Olomouc Czech Repub 152: 251-258.

[2] Differential Effect of Iron and Myelin on Susceptibility MRI in the Substantia Nigra. Radiology 301: 682-691.

[3] Kozel J, Skoloudik D, Ressner P et al (2023) Echogenicity of Brain Structures in Huntington's Disease Patients Evaluated by Transcranial Sonography - Magnetic Resonance Fusion Imaging using Virtual Navigator and Digital Image Analysis. Ultraschall Med 44: 495-502.

**Supplementary Table 1** Comparison of results of left and right substantia nigra parameters within each group.

| Parkinson’s disease | Left side | Right side | *t/U* | *P* |
| --- | --- | --- | --- | --- |
| aSNH (cm^2^) | 0.21 (0,0.30) | 0.05 (0,0.19) | 4.85 | <0.001^*^ |
| Pixel count of SN1 | 1196.79±229.12 | 1179.55±230.87 | 0.68 | 0.498^#^ |
| GSM of SN1 | 25.11±6.87 | 20.06±9.12 | 5.67 | <0.001^#^ |
| Pixel count of SN2 | 1198.19±248.37 | 1152.94±245.17 | 1.24 | 0.218^#^ |
| GSM of SN2 | 28.45±7.31 | 23.07±9.69 | 4.13 | <0.001^#^ |
| Pixel count of SN3 | 1076.73±203.41 | 1056.02±211.09 | 0.90 | 0.367^#^ |
| GSM of SN3 | 24.56±1.30 | 21.05±9.45 | 3.04 | 0.003^#^ |
| Multiple system atrophy-parkinsonian type | Left side | Right side | *t/U* | *P* |
| aSNH (cm^2^) | 0 (0,0.19) | 0 (0,0.08) | 1.48 | 0.138^*^ |
| Pixel count of SN1 | 1138.83±224.14 | 1119.71±224.74 | 0.50 | 0.622^#^ |
| GSM of SN1 | 18.63±6.78 | 19.25±8.97 | 0.47 | 0.642^#^ |
| Pixel count of SN2 | 1135.19±245.89 | 1096.68±261.54 | 0.57 | 0.571^#^ |
| GSM of SN2 | 23.18±7.52 | 23.70±9.73 | 0.23 | 0.821^#^ |
| Pixel count of SN3 | 1028.32±202.81 | 959.78±213.60 | 1.90 | 0.030^#^ |
| GSM of SN3 | 20.55±9.39 | 19.20±9.68 | 0.84 | 0.403^#^ |
| Healthy control | Left side | Right side | *t/U* | *P* |
| aSNH (cm^2^) | 0 (0,0.18) | 0 (0,0.07) | 1.90 | 0.058^*^ |
| Pixel count of SN1 | 1191.21±236.25 | 1185.46±213.66 | 0.20 | 0.845^#^ |
| GSM count of SN1 | 18.31±5.75 | 16.77±7.20 | 1.82 | 0.070^#^ |
| Pixel count of SN2 | 1255.94±257.23 | 1231.98±231.16 | 0.46 | 0.649^#^ |
| GSM of SN2 | 22.06±6.98 | 20.76±8.00 | 0.82 | 0.417^#^ |
| Pixel count of SN3 | 1067.87±197.37 | 1059.86±193.42 | 0.32 | 0.753^#^ |
| GSM of SN3 | 20.16±7.74 | 17.44±6.66 | 2.90 | 0.004^#^ |

Note：

*t/U*: *t* or *U* statistic as appropriate to the test;

^*^Mann-Whitney U test;

^#^unpaired independent t test;

aSNH was expressed as median (interquartile range), while other parameters were presented as mean ± standard deviation.

aSNH: area of substantia nigra hyperechogenicity; SN, substantia nigra; GSM, grayscale median
